# Outcomes of allogeneic hematopoietic cell transplantation in patients with carbapenem-resistant organisms infection: a propensity score-matched analysis

**DOI:** 10.3389/frtra.2026.1818037

**Published:** 2026-05-15

**Authors:** Abeer Yaseen, Teeba Mubaydeen, Hazem Al-qaraleh, Ahmad Otoom, Shadee Shaban, Hazim Mesmar, Albatoul Alomoush, Waleed Da'Na, Khalid Halahleh, Husam Abu Jazar, Salwa Saadeh, Mohammad Ma'koseh, Abdulrahman Shamieh, Susan Kullab, Osama Abu Ata, Joud Jarrah, Zaid Abdel Rahman

**Affiliations:** 1 Department of Internal Medicine, King Hussein Cancer Center, Amman, Jordan; 2 University of Jordan, School of Medicine, Amman, Jordan; 3 Office of Scientific Affairs and Research, King Hussein Cancer Center, Amman, Jordan

**Keywords:** allogeneic hematopoietic cell transplantation, carbapenem-resistant organisms, non-relapse mortality, overall survival, propensity score-matched analysis

## Abstract

**Background:**

Carbapenem-resistant organisms (CRO) cause significant morbidity and mortality, with a rising incidence worldwide. Immunosuppression, prolonged hospitalization, and extensive use of broad-spectrum antibiotics make allogeneic hematopoietic cell transplant (alloHCT) recipients particularly susceptible. This study evaluated the impact of peri-transplant CRO infections on alloHCT outcomes.

**Methods:**

We conducted a retrospective analysis of adult alloHCT recipients between 2015 and 2024. CRO infection was defined as occurring within 12 months of alloHCT. Using 1:3 propensity score matching, patients with CRO were matched to controls by age, sex, year of transplant, stem cell source, donor type, and diagnosis. Overall survival (OS) and non-relapse mortality (NRM) were analyzed using Kaplan–Meier methods and Cox proportional hazards models.

**Results:**

A total of 171 patients were included: 43 with CRO and 128 controls; 53.4% of CRO cases occurred in the last 3 years. Median age was 35 years, 66.7% were male, and 89.5% underwent transplant for malignant disease. Matched-related donors were used in 79.5%, peripheral blood stem cells in 88.3%, and myeloablative conditioning in 70.2%. Median follow-up was 27 months. CRO patients had higher ICU admission rates (30.2% vs. 17.2%, *p* = 0.067) and lower 1-year OS (46.5% vs. 72.7%, *p* = 0.002). On multivariate analysis, CRO infection was independently associated with worse OS, while engraftment predicted improved OS and lower NRM.

**Conclusions:**

CRO infections remain an increasing challenge in alloHCT and are associated with higher ICU admission, increased NRM, and inferior survival, highlighting the need for improved prevention and management strategies

## Introduction

Allogeneic hematopoietic cell transplantation (alloHCT) has been the mainstay treatment for a variety of malignant and non-malignant hematologic conditions (1). It offers the potential for curative outcomes, particularly in conditions unresponsive to standard treatments. However, the success of alloHCT is sometimes shadowed by its substantial risk of complications, which include graft-vs.-host disease (GVHD), relapses, and infectious complications. Among these, infections remain a major concern due to the profound and often prolonged immunosuppression associated with preparative conditioning regimens, post-transplant immunosuppressive therapies to prevent GVHD, and the delayed immune reconstitution, especially during the first 100 days post-transplant (2–4). Furthermore, alloHCT recipients often require prolonged indwelling intravenous catheters, extended hospitalization, and long-term antibiotic therapy, creating a conducive environment for acquiring infections with multidrug-resistant organisms (MDRO) (5).

MDROs, including carbapenem-resistant organisms (CROs), represent a significant challenge in modern healthcare due to the high morbidity and mortality rates, the growing global prevalence of antimicrobial resistance and limited available treatment options (6–12). CROs have been identified by the Centers for Disease Control and Prevention (CDC) in the United States and the World Health Organization (WHO) as a critical priority in clinical health (13, 14). These organisms often complicate post-transplant recovery, leading to prolonged hospitalization, increased intensive care units (ICU) stays, increased healthcare costs, and poor clinical outcomes.

Despite the global attention to CROs, data from the Middle East, and particularly from Jordan, remain limited. Our study aims to evaluate the incidence and clinical implications of peri-alloHCT CRO infections among patients undergoing alloHCT at a tertiary cancer center in, Jordan.

## Methods

### Study design and data collection

We conducted a retrospective cohort study of adult patients (≥18 years) who underwent alloHCT at King Hussein Cancer Center (KHCC) between 2015 and 2024. Baseline demographic, transplant, and outcome data were extracted from KHCC's local HCT registry and supplemented through review of electronic medical records, including details on complications and causes of death.

### Definitions

Indications for alloHCT were in concordance with recommendations from the American Society of Transplantation and Cellular Therapies (ASTCT) (15). Conditioning intensity was defined according to the criteria proposed by the Center for International Blood and Marrow Transplant Research (CIBMTR) (16). Conventional GVHD prophylaxis was used in most cases (i.e., calcineurin inhibitor in combination with methotrexate), for patients with haploidentical donors post-transplant cyclophosphamide (PTCy) was administered. Overall survival (OS) was defined as the time from the date of HCT until death or last observation alive. Relapse was defined as the reappearance of blasts in the blood or bone marrow (>5%) or any extra-medullary site after a complete response (CR) for malignant disorders. Non-Relapse Mortality (NRM) was defined as death without relapse after alloHCT.

Carbapenem resistance was defined as resistance to at least one carbapenem antibiotic (e.g., ertapenem, meropenem, or imipenem) or the production of carbapenemase enzymes as detected on laboratory testing (17). Molecular genotyping for specific carbapenemase genes (e.g., NDM, OXA-48, VIM, IMP, KPC) was not performed at our center during the study period due to resource limitations. Therefore, resistance was determined phenotypically based on susceptibility testing to at least one carbapenem or documented carbapenemase production according to standard laboratory protocols. Carbapenem-resistant organisms (CRO) infections were defined as infections with one or more of the following microorganisms: (1) Enterobacteriaceae family: *Escherichia coli, Klebsiella, Enterobacter, Citrobacter, Salmonella and Shigella;* (2) Morganellacaeae family*: Proteus, Morganella, and Providencia;* (3) Others*: Pseudomonas, Serratia, Hafnia, and Yersinia.* Patients were included if they developed a CRO infection within 12 months of alloHCT.

Antibiotics were classified into 3 classes according to the WHO AWaRe classification (18): (1) Access: narrow spectrum/low potential for resistance (e.g., amoxicillin, doxycycline, and metronidazole), (2) Watch: broader spectrum/higher potential for resistance (e.g., ciprofloxacin, piperacillin-tazobactam, Azithromycin, and ceftriaxone) and (3) Reserve: last-resort antibiotics to use very selectively (e.g., Colistin, carbapenems, and ceftazidime-avibactam). Empiric antimicrobial therapy in neutropenic patients is guided by local antibiograms, patient risk stratification, and MDRO surveillance results. Routine perianal and nasal swab cultures for MDRO colonization are performed upon admission to the transplant floor. In high-risk febrile neutropenia, broad-spectrum Watch- or Reserve-class agents (commonly piperacillin-tazobactam or a carbapenem) are initiated empirically. Escalation to Reserve-class antibiotics (most frequently colistin-based regimens, often in combination with meropenem, or ceftazidime-avibactam when indicated and available) is performed in cases of clinical instability, preliminary microbiologic data, or known colonization status. De-escalation occurs once final culture identification and susceptibility results are available. Of note, ceftazidime-avibactam became available at our center in 2021 and was used as targeted therapy in eligible cases during the later part of the study period.

### Statistical analysis

Continuous variables were summarized using the sample median and range. Categorical variables were summarized with numbers and percentages. The cumulative incidence of death after transplantation was estimated using the Kaplan–Meier method. The cumulative incidence of NRM was estimated, accounting for the competing risk of relapse, using the Kaplan–Meier method. Associations with outcomes were evaluated using univariate (i.e., unadjusted) and multivariable Cox proportional hazards regression models, where the cause-specific hazard of the given outcome was modeled. Hazard ratios (HRs) and 95% confidence intervals (CIs) were estimated. CRO infection was forced into the multivariate analysis (MVA) model as the primary variable of interest. Additional covariates for MVA were selected based on clinical relevance, statistical significance in univariate analysis (UVA), and their potential to confound the association between CRO infection and survival outcomes. To reduce the risk of overfitting, the number of variables included in each model was limited according to the commonly accepted rule of one variable per 10 events. *P* values < 0.05 were considered statistically significant, and all statistical tests were two-sided. All statistical analysis was performed using SPSS v.29.

A 1:3 propensity score matching was used to balance variables between patients with and without CRO infection (19–21). Propensity scores were estimated using a logistic regression model that included the following variables: age at transplant, gender, stem cell source, donor type, and diagnosis. We selected a 1:3 ratio (rather than 1:1) to maximize statistical power and precision, given the relatively small number of patients with CRO infection (*n* = 43) compared with the total pool of 486 alloHCT recipients. This approach allowed inclusion of 128 well-matched controls while preserving good balance between groups. Balance between groups was assessed using absolute standardized mean differences (SMDs), with values <0.1 considered optimal and <0.2 acceptable. Details of the propensity score matching procedure are provided in Supplementary Table S1. Patient selection, exclusions, and the matching process are illustrated in Supplementary Figure S1.

OS and NRM were compared between the matched groups using Kaplan–Meier survival analysis and Cox proportional hazards models. Conditioning intensity was categorized as myeloablative or reduced-intensity according to CIBMTR criteria and was included in the propensity score matching as well as in the univariate and multivariate Cox models.

This study was approved by the Institutional Review Board (IRB) at KHCC (Study #23 KHCC 172) and conducted according to the declaration of Helsinki.

## Results

### Patient characteristics

During the study period, 486 patients underwent alloHCT at KHCC. Among these, 56 patients were identified as having a CRO infection, of whom 43 met inclusion criteria for peri-transplant infection. Patients with incomplete medical records or CRO infections beyond the 12-month peri-transplant window, or with asymptomatic colonization without a documented clinically evident infection were excluded. A propensity score-matched control cohort comprising of 128 patients without a documented CRO infection was selected in a 1:3 ratio, post-matching, all covariates were well balanced with absolute standardized mean differences <0.2 for all variables. The final analytic cohort consisted of 171 patients. The median age at alloHCT for the entire cohort was 37 years (range: 18–64), and the majority were males (*N* = 114, 66.7%). Most patients (*N* = 153, 89%) underwent alloHCT for a malignant disorder with acute myelogenous leukemia (AML) being the most common indication (*N* = 77, 45%). The majority (*N* = 136, 79.5%) of donors were matched siblings (MSD), while 33 (19.3%) patients had haploidentical donors and 2 patients (1%) received stem cells from a matched unrelated donor (MUD). Peripheral blood was the predominant graft source (*n* = 151, 88.3%), and myeloablative conditioning was used in 120 (79.5%) patients. Table 1 provides a detailed overview of baseline clinical and transplant characteristics.

**Table 1:** Patients’ baseline and HCT characteristics.

Variable	Entire cohort (*N* = 171)	CRO infection (*N* = 43)	No CRO infection (*N* = 128)
Age at HCT, Years			
Median (Range)	35 (18–64)	32 (19–57)	35.5 (18–64)
Gender, *N* (%)			
Male	114 (66.7)	29 (67.4)	85 (66.4)
Female	62 (33.3)	14 (32.6)	43 (33.6)
HCT Year, *N* (%)			
2015–2019	93 (54.4)	15 (34.9)	78 (60.9)
2020–2024	78 (45.6)	28 (65.1)	50 (39.1)
Dx to HCT, Months			
Median (Range)	8 (0–509)	7 (2–509)	8 (0–370)
Disease, *N* (%)			
Malignant	153 (89.5)	37 (86)	116 (90.6)
Non-Malignant	18 (10.5)	6 (14)	12 (9.4)
Indication for Transplant, *N* (%)			
Acute Myeloid Leukemia	77 (45)	20 (46.5)	57 (44.5)
Acute Lymphoblastic Leukemia	36 (21.1)	10 (23.3)	26 (20.3)
Myelodysplastic Syndrome	17 (9.9)	3 (7)	14 (10.9)
Bone Marrow Failure Syndrome	18 (10.5)	6 (14)	12 (9.4)
Myelofibrosis	8 (4.7)	1 (2.3)	7 (5.5)
Non-Hodgkin Lymphoma	5 (2.9)	2 (4.7)	3 (3.2)
Hodgkin Lymphoma	7 (4.1)	1 (2.3)	6 (4.7)
Chronic Myeloid Leukemia	2 (1.2)	0 (0)	2 (1.6)
Biphenotypic Leukemia	1 (0.6)	0 (0)	1 (0.8)
Conditioning Intensity, *N* (%)			
RIC	51 (29.8)	15 (34.9)	36 (28.1)
Myeloablative	120 (70.2)	28 (65.1)	92 (71.9)
Donor Relation, *N* (%)			
MSD	136 (79.5)	30 (69.8)	106 (82.8)
Haploidentical	33 (19.3)	12 (27.9)	12 (16.4)
MUD	2 (1.2)	1 (2.3)	1 (0.8)
Transplant Source, *N* (%)			
Bone Marrow	19 (11.1)	6 (14)	13 (10.2)
Peripheral Blood	151 (88.3)	37 (86)	114 (89.1)
Both	1 (0.6)	0 (0)	1 (0.8)
GVHD Prophylaxis, *N* (%)			
CNI + MTX	110 (64.3)	26 (60.5)	84 (65.6)
CNI + MMF	26 (15.2)	4 (9.3)	22 (17.2)
PTCy-based	35 (20.5)	13 (30.2)	22 (17.2)
Recipient CMV Status, *N* (%)			
Positive	171 (100)	43 (100)	128 (100)

CRO, Carbapenem-resistant organism; HCT, Hematopoietic cell transplantation; RIC, Reduced-intensity conditioning; MSD, Matched-sibling donor; MUD, Matched-unrelated donor; GVHD, Graft vs. host disease; CNI, Calcineurin inhibitor; MTX, Methotrexate; MMF, Mycophenolate mofetil; PTCy, Post-transplant cyclophosphamide; CMV, Cytomegalovirus.

### Epidemiology and characteristics of CRO infections

Among the 43 patients with CRO infections, 7 (16.3%) had prior documented colonization.

A total of 47 infections were documented: 9 (20.9%) patients were diagnosed with a CRO infection before alloHCT, 30 (69.8%) patients after alloHCT, and 4 (9.3%) patients had documented infections before and after alloHCT, the majority of post-HCT infections occurred within the first 100 days (Table 2). Among the 34 patients with post-HCT CRO infections (4 had before and after HCT infection), 22 (64.7%) occurred within the first 100 days post-HCT (early infection) and 12 (35.3%) occurred after day +100 (late infection).

**Table 2:** Characteristics of carbapenem-resistant organism (CRO) infections.

Characteristic	*N* (%)
Culture Isolates, *N* (%)	
Escherichia coli	27 (57.4)
Klebsiella pneumonia	10 (21.27)
Enterobacter	2 (4.25)
Serratia marcescens	1 (2.12)
Acinetobacter baumannii	1 (2.12)
Pseudomonas aeruginosa	5 (10.63)
Both E. coli and A. baumannii	1 (2.12)
Prior colonization, *N* (%)	
Yes	7 (16.3)
No	36 (83.7)
Time of infection, *N* (%)	
Before HCT	9 (18.6)
After HCT	30 (69.8)
Before and After HCT	4 (9.3)
Time of infection post-HCT (early vs. late), *N* (%)	34
≤ 100 days post-HCT	22 (64.7)
> 100 days post-HCT	12 (35.3)
Sample source, *N* (%)	
Blood stream	26 (55.31)
Urinary tract	11 (23.4)
Skin and soft tissue	5 (10.63)
Lung	3 (6.38)
Upper respiratory tract/Sinus	2 (4.25)
CVC present at time of infection, *N* (%)	
Yes	27 (62.8)
No	16 (37.2)
Need for ICU admission, *N* (%)	
Yes	16 (37.2)
No	27 (62.8)
Empiric antimicrobial therapy class, *N* (%)	
Access	1 (2.12)
Watch	25 (53.19)
Reserve	21 (44.68)
Adjusted antimicrobial class, *N* (%)	
Access	2 (4.25)
Watch	10 (21.27)
Reserve	35 (74.46)

CVC, central venous catheter; ICU, intensive care unit; HCT, hematopoietic cell transplantation.

Most of the infections (53.4%) were diagnosed in the last 3 years of the study period, between 2021 and 2023.

**Figure 1:**
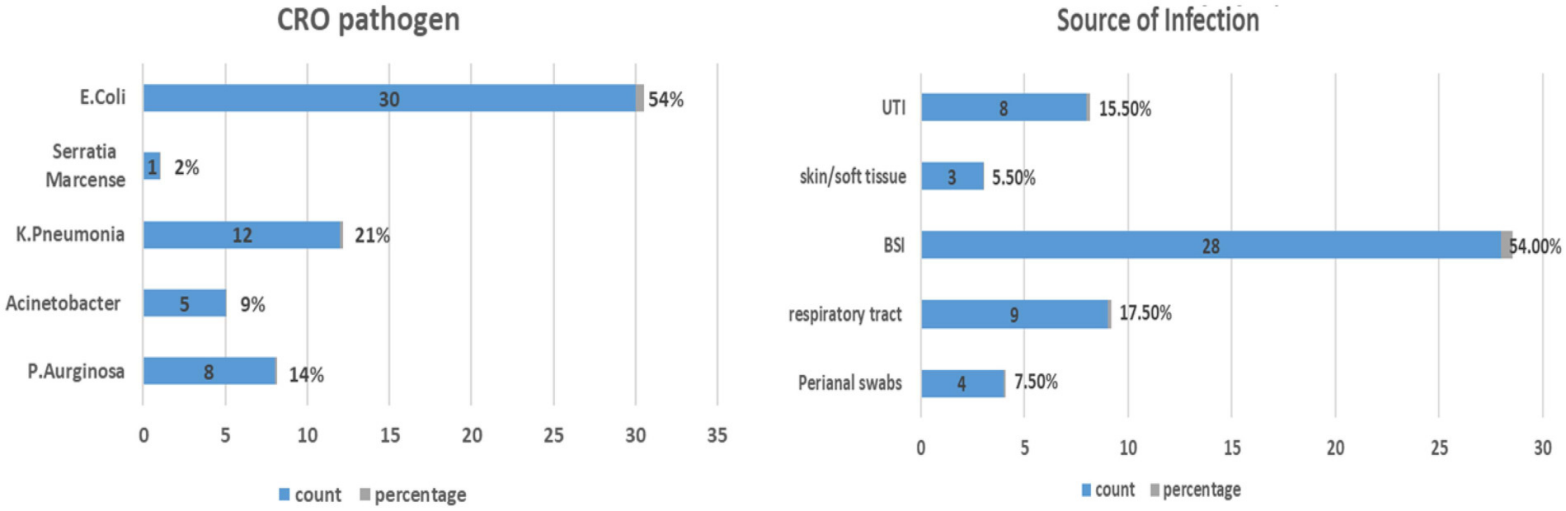
Distribution of CRO pathogens and sources of infection.

Escherichia coli was the most common isolated pathogen, accounting for 57.4% (*N* = 27) of infections, followed by Klebsiella pneumonia (*N* = 10, 21.27%), Pseudomonas aeruginosa (*N* = 5, 10.63%), and Enterobacter (*N* = 2, 4.25%). One infection was caused by Serratia marcescens and Acinetobacter baumannii each, and one patient had two isolates involving E. coli and A. baumannii. (Figure 1).

The most common source of infection was the bloodstream, accounting for 55.31% (*N* = 26) of infections, followed by the urinary tract (*N* = 11, 23.4%), skin and soft tissue (*N* = 5, 10.63%), and respiratory tract (*N* = 5). (Figure 1).

Regarding treatment with antimicrobial agents, empirically initiated antibiotics were predominantly classified as “Watch” (*n* = 25, 53.2%), with “Reserve” antibiotics used in 21 cases (44.7%) and “Access” antibiotics in one case. After speciation and availability of culture results, escalations from “Watch” to “Reserve” antibiotics occurred in 16 patients required, while 3 patients had de-escalations (2 from “Reserve” to “Watch” and 1 from “Watch” to “Access”). Reserve-class antibiotics were ultimately used in 35 infections (74.5%), most commonly colistin-based regimens (often in combination with meropenem). Ceftazidime-avibactam was administered to 13 patients (10 for post-HSCT infections and 3 for pre-HSCT infections), primarily as targeted therapy.

### Survival outcomes

**Figure 2:**
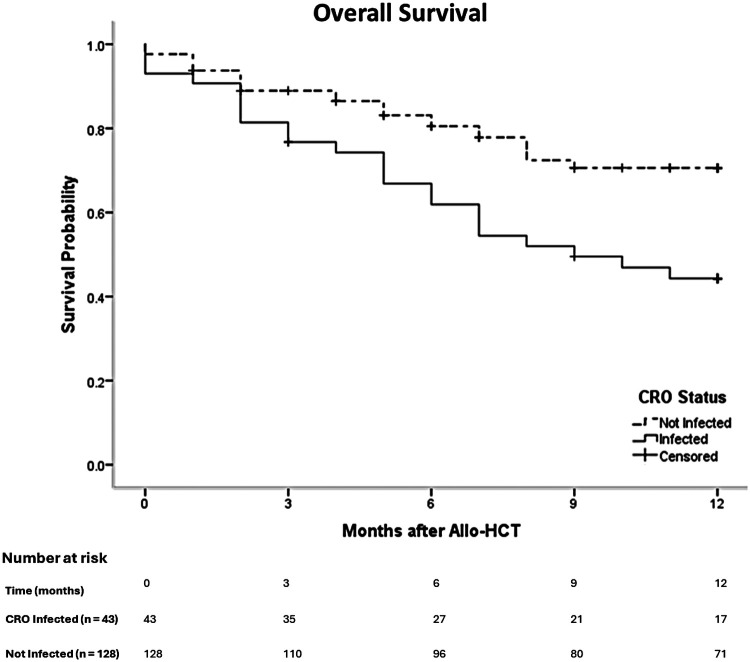
Overall survival stratified by CRO infection Status.

At a median follow-up of 27 months (range: 17–37), 1-year overall survival (OS) was significantly inferior in the CRO cohort compared to matched controls (46.5% vs. 72.7%, *p* = 0.002). Median OS in the CRO group was 9 months (95% CI: 4.6–13.3), while it was not reached in the control group (Figure 2). Both early and late CRO infections post-HCT were associated with very poor outcomes, although formal statistical comparison was not performed due to small subgroup sizes.

**Figure 3:**
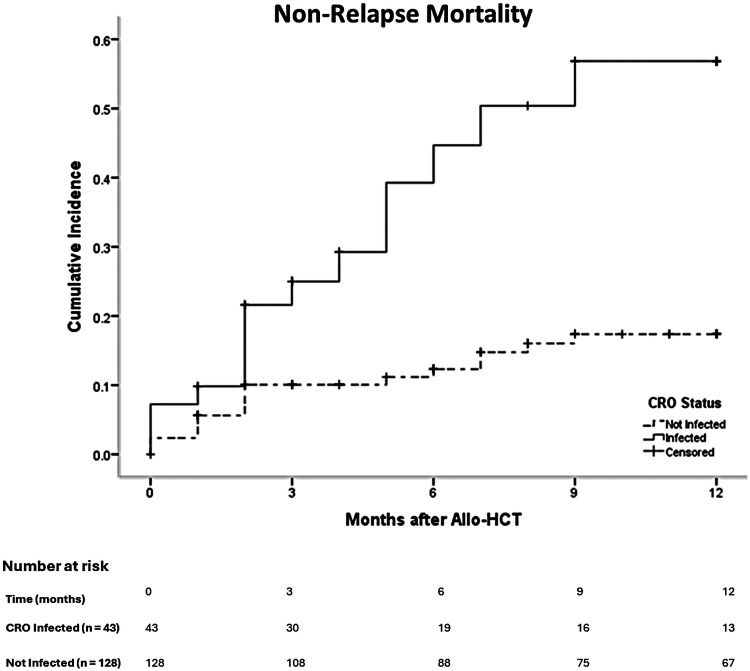
Non-Relapse mortality stratified by CRO infection Status.

The 1-year cumulative incidence of non-relapse mortality (NRM) was markedly elevated in the CRO group (34.9% vs. 14.1%, *p* = 0.001) (Figure 3).

### Univariate and multivariate analyses

On univariate analysis, CRO infection was associated with lower over survival (HR: 2.32, 95% CI: 1.46–3.69, *p* < 0.001). Additional adverse prognostic factors included presence of malignancy (HR: 7.39, 95% CI: 1.81–30.1, *p* = 0.005), haploidentical donor source (HR: 2.65, *p* < 0.001), use of PBSC (HR: 4.85, 95% CI: 1.53–15.3, *p* = 0.007), and PTCy-based GVHD prophylaxis (HR: 2.78, 95% CI: 1.65–4.66, *p* < 0.001). Successful engraftment (HR: 0.16, 95% CI: 0.07–0.36, *p* < 0.001) and RIC (HR: 0.62, 95% CI: 0.4–0.97, *p* = 0.039) were associated with improved OS.

Multivariate analysis (MVA) confirmed CRO infection as an independent adverse predictor of OS (HR: 1.87, 95% CI: 1.13–3.09, *p* = 0.015), while engraftment retained its protective association (HR: 0.20, 95% CI: 0.08–0.48, *p* < 0.001). (Table 3).

**Table 3:** Univariate and multivariate analysis for overall survival (OS).

Univariate analysis				
Variable	Hazard ratio	Lower 95% CI	Upper 95% CI	*P* Value
CRO infection	2.324	1.462	3.694	<.001
Gender (Male)	0.791	0.495	1.265	0.328
Malignancy	7.39	1.814	30.106	0.005
Donor relation:				
MRD	Reference			
Haploidentical	2.651	1.598	4.395	<.001
MUD	1.218	0.168	8.814	0.846
Stem cell source:				
BM	Reference			
PSC	4.85	1.53	15.378	0.007
Conditioning Intensity (RIC)	0.626	0.401	0.977	0.039
GVHD prophylaxis:				
CNI + MTX	Reference			
CNI + MMF	1.32	0.736	2.365	0.352
PTCy-based	2.781	1.657	4.668	<.001
Engraftment	0.162	0.074	0.357	<.001
Time from diagnosis to transplant	1.003	0.999	1.006	0.125
Age at transplant	1.018	1.000	1.036	0.052
Multivariate analysis				
	Hazard Ratio	Lower 95% CI	Upper 95% CI	*P* Value
CRO infection	1.867	1.127	3.092	0.015
Engraftment	0.205	0.088	0.480	<.001
GVHD prophylaxis:				
CNI MTX	Reference			
CNI + MMF	1.286	0.676	2.449	0.444
PTCy-based	2.253	0.307	16.558	0.425
Donor relation:				
MRD	Reference			
Haploidentical	1.009	0.135	7.575	0.993
MUD	1.051	0.139	7.960	0.962
Age at transplant	1.019	0.999	1.039	0.067

For NRM, univariate analysis identified CRO infection (HR: 2.43, 95% CI: 1.27–4.64, *p* = 0.007), haploidentical donor source (HR: 3.03, 95% CI: 1.50–6.07, *p* = 0.002), older age (HR: 1.04 per year, 95% CI: 1.01–1.06, *p* = 0.004), and PTCy-based GVHD prophylaxis (HR: 3.43, 95% CI: 1.67–7.02, *p* < 0.001) as significant predictors of increased mortality. Engraftment again demonstrated a protective effect (HR: 0.078, 95% CI: 0.03–0.18, *p* < 0.001).

On MVA for NRM, CRO infection remained an independent risk factor (HR: 2.06, 95% CI: 1.01–4.21, *p* = 0.048), alongside age (HR: 1.05, 95% CI: 1.02–1.07, *p* < 0.001), while successful engraftment continued to be significantly associated with reduced NRM (HR: 0.084, 95% CI: 0.03–0.21, *p* < 0.001). (Table 4).

**Table 4:** Univariate and multivariate analysis for Non-relapse mortality (NRM).

Univariate analysis				
Variable	Hazard ratio	Lower 95% CI	Upper 95% CI	*P* Value
CRO Infection	2.432	1.273	4.647	0.007
Gender (Reference: Male)	0.594	0.292	1.209	0.151
Malignancy	3.284	0.791	13.632	0.102
Donor relation:				
MRD	Reference			
Haploidentical	3.025	1.506	6.078	0.002
MUD	2.658	0.36	19.632	0.338
Stem cell source:				
BM	Reference			
PSC	6.729	0.925	48.982	0.060
Both	0	0		0.981
Conditioning intensity (Reference: Reduced)	0.577	0.309	1.077	0.084
GVHD Prophylaxis:				
CNI + MTX	Reference			
CNI + MMF	1.65	0.73	3.727	0.229
PTCy-based	3.43	1.676	7.021	<.001
Engraftment	0.078	0.033	0.18	<.001
Time from diagnosis to transplant	1.004	1	1.007	0.045
Age at transplant	1.037	1.011	1.062	0.004
Multivariate analysis				
	Hazard Ratio	Lower 95% CI	Upper 95% CI	*P* Value
CRO infection	2.060	1.008	4.212	0.048
Engraftment	0.084	0.033	0.216	<.001
Age at transplant	1.049	1.021	1.078	<.001

Notably, ICU admission was more frequent among patients with CRO infection compared to matched controls (30.2% vs. 17.2%, *p* = 0.067). These admissions occurred concurrently with or shortly after CRO diagnosis in the majority of cases and were primarily driven by sepsis or organ failure related to the resistant organism. ICU admission was not included in the final multivariable models due to strong collinearity with CRO infection status. Nevertheless, CRO infection remained independently associated with inferior OS and higher NRM after adjustment for other covariates.

## Discussion

In this analysis of outcomes of alloHCT recipients at a tertiary cancer center in Jordan, we found that peri-transplant CRO infections were associated with significantly inferior OS and increased NRM compared with compared with propensity score-matched controls. CRO infectionsremained an independent adverse prognostic factor in multivariate analyses. These findings underscore the critical impact of antimicrobial resistance on post-transplant outcomes in resource-limited settings.

Our results are consistent with prior studies from western cohorts demonstrating that CRO infections significantly increase post-transplant morbidity and mortality (12, 22, 23). Bloodstream infections predominated, in line with other cohorts where prolonged neutropenia, mucosal barrier injury, and central venous catheters drive invasive infections (24, 25). E. coli and Klebsiella were the most common isolates, mirroring global trends (26–28), though a higher proportion of Pseudomonas aeruginosa in our series may reflect local antibiotic prescribing patterns.

More than half of infections occurred in the last 3 years of the study period (2021 and 2023) (), raising concern for an escalating burden. This may be related to increased broad-spectrum antibiotic use during the post COVID-19 era (29, 30), greater reliance on haploidentical transplantation (31), and evolving local resistance patterns. These observations emphasize the need for ongoing surveillance and strengthened preventive strategies in transplant centers.

CRO infections remain particularly difficult to manage due to limited treatment options. Over 70% of affected patients required Reserve-class antibiotics, most commonly colistin-based regimens that areassociated with significant toxicity and risk of further resistance (32, 33). The frequent escalation from Watch- to Reserve-class agents highlights the difficulty of establishing empiric therapy selection in this setting.

Transplant-related factors such as haploidentical donors (34), were linked to inferior outcomes, consistent with published reports associating it with higher infectious risk. However, interpretation should be cautious due to potential confounding and small subgroup sizes. In contrast, successful engraftment emerged as a strong protective factor, emphasizing the importance of rapid immune recovery in reducing infection-related mortality (35, 36). Although ICU admission was more common in the CRO cohort, it largely occurred as a consequence of severe CRO infection rather than as an independent preceding factor, reinforcing the direct clinical impact of these resistant infections.

In our cohort, the association between PBSC use and inferior outcomes may be explained by our institutional practice, whereby bone marrow grafts were mainly utilized for aplastic anemia while PBSC was predominantly used for malignant indications, thus introducing confounding by indication rather than a direct graft-source effect.

Increasing age at transplant was independently associated with higher NRM in MVA. This observation is consistent with prior studies showing that older patients have higher vulnerability to treatment-related toxicity, delayed immune recovery, and infectious complications after alloHCT (37, 38).

Routine perianal and nasal swab surveillance for MDRO colonization is performed at our center upon admission to the transplant floor. Although colonization is a well-recognized risk factor for subsequent invasive infection (39), only a minority of patients in this cohort had documented colonization prior to infection, limiting further subgroup analysis. Active screening enables timely initiation of targeted therapy, while preventive strategies such as infection control measures, antimicrobial stewardship, and individualized antibiotic plans remain critical. Emerging approaches like fecal microbiota transplantation may help reduce MDRO colonization, though their role in transplant recipients is still investigational (40, 41).

In the last decade, several new antimicrobials have expanded the treatment options for multidrug-resistant Gram-negative infections, including CRE. *β*-lactam/*β*-lactamase inhibitor combinations such as ceftazidime–avibactam (42), meropenem–vaborbactam (43), and imipenem–relebactam (44) have become important agents against KPC- and OXA-producing organisms, the most prevalent carbapenemases worldwide. Investigational agents such as zidebactam (45, 46), taniborbactam (47), and nacubactam (48, 49) are under evaluation, and bacteriophage therapy has emerged as a potential adjunct (50, 51), although robust clinical trial data in transplant recipients remain limited.

In the Middle East, including Jordan, OXA-48-like and NDM enzymes predominate and frequently co-exist in carbapenem-resistant Enterobacterales (52, 53). This pattern explains the frequent reliance on colistin-based regimens in our cohort and the selective use of ceftazidime-avibactam, which was primarily employed for suspected OXA-48 producers. While ceftazidime-avibactam retains excellent activity against OXA-48-like enzymes, it has limited or no activity against NDM-producing strains unless combined with aztreonam. The lack of routine molecular genotyping in our centre during the study period therefore limits both mechanistic insights and the precision with which newer *β*-lactam/*β*-lactamase inhibitor combinations can be applied.

our knowledge, our study represents one of the first reports from the Middle East specifically evaluating the clinical impact of CRO infections in alloHCT recipients. However, several limitations should be acknowledged. The study has a retrospective, single-center design with a relatively small sample size in the CRO group (*n* = 43), limiting statistical power for subgroup analyses. Despite propensity score matching on key variables, residual confounding cannot be fully excluded, particularly due to the lack of data on acute and chronic GVHD, HCT-CI, Disease Risk Index (DRI), cumulative immunosuppression, steroid exposure, and detailed prior antibiotic history. We defined peri-transplant CRO infection within a broad 12-month window around alloHCT, which combines biologically distinct phases including pre-engraftment neutropenia, early post-engraftment, and late GVHD-related infections. Molecular genotyping for carbapenemase genes was not performed due to resource constraints, with resistance determined phenotypically only.

Future research should prioritize prospective, multicenter studies to better define the epidemiology of CRO infections in transplant recipients across diverse regions. Clinical trials evaluating novel antimicrobial agents in the transplant setting are urgently needed, as are real-world studies assessing their efficacy and toxicity in LMIC populations. At the institutional level, our findings support the implementation of robust infection control programs, judicious antibiotic stewardship guided by local antibiograms, and targeted prophylactic strategies for colonized patients.

## Conclusion

CRO infections are a significant predictor of poor outcomes in alloHCT recipients, as demonstrated by their association with higher NRM, reduced OS and increased ICU admissions. These findings underscore the urgent need for effective preventive measures, antibiotic stewardship programs, and innovative therapeutic strategies to mitigate the impact of CRO infections in this high-risk population.

## Data Availability

The raw data supporting the conclusions of this article will be made available by the authors, without undue reservation.

## References

[1] Majhail NS Farnia SH Carpenter PA Champlin RE Crawford S Marks DI Indications for autologous and allogeneic hematopoietic cell transplantation: guidelines from the American society for blood and marrow transplantation. Biol Blood Marrow Transplant. (2015) 21:1863–9. 10.1016/j.bbmt.2015.07.032 26256941 PMC4830270

[2] Cao W Zhang J Bian Z Li L Zhang S Qin Y Active screening of intestinal colonization of carbapenem-resistant Enterobacteriaceae for subsequent bloodstream infection in allogeneic hematopoietic stem cell transplantation. Infect Drug Resist. (2022) 15:5993–6006. 10.2147/IDR.S387615 36262593 PMC9576326

[3] For the Infectious Diseases Working Party EBMT. Styczyński J Tridello G Koster L Iacobelli S van Biezen A van der Werf S Death after hematopoietic stem cell transplantation: changes over calendar year time, infections and associated factors. Bone Marrow Transplant. (2020) 55:126–36. 10.1038/s41409-019-0624-z 31455899 PMC6957465

[4] Akhmedov M. Infectious complications in allogeneic hematopoietic cell transplant recipients: review of transplant-related risk factors and current state of prophylaxis. Clin Transplant. (2021) 35:e14172. 10.1111/ctr.14172 33247497

[5] César-Arce A Volkow-Fernández P Valero-Saldaña LM Acosta-Maldonado B Vilar-Compte D Cornejo-Juárez P. Infectious complications and multidrug-resistant Bacteria in patients with hematopoietic stem cell transplantation in the first 12 months after transplant. Transplant Proc. (2017) 49:1444–8. 10.1016/j.transproceed.2017.03.081 28736021

[6] Heidenreich D Kreil S Nolte F Hofmann WK Miethke T Klein SA. Multidrug-resistant organisms in allogeneic hematopoietic cell transplantation. Eur J Haematol. (2017) 98:485–92. 10.1111/ejh.12859 28135011

[7] Satlin MJ Walsh TJ. Multidrug-resistant Enterobacteriaceae, *P seudomonas aeruginosa*, and vancomycin-resistant *Enterococcus* : three major threats to hematopoietic stem cell transplant recipients. Transpant Infect Dis. (2017) 19:e12762. 10.1111/tid.12762 PMC5745272 28815897

[8] Girmenia C Rossolini GM Piciocchi A Bertaina A Pisapia G Pastore D Infections by carbapenem-resistant Klebsiella pneumoniae in SCT recipients: a nationwide retrospective survey from Italy. Bone Marrow Transplant. (2015) 50:282–8. 10.1038/bmt.2014.231 25310302

[9] Kim SB Min YH Cheong JW Kim JS Kim SJ Ku NS Incidence and risk factors for carbapenem- and multidrug-resistant Acinetobacter baumannii bacteremia in hematopoietic stem cell transplantation recipients. Scand J Infect Dis. (2014) 46:81–8. 10.3109/00365548.2013.857042 24325335

[10] Pouch SM Satlin MJ. Carbapenem-resistant Enterobacteriaceae in special populations: solid organ transplant recipients, stem cell transplant recipients, and patients with hematologic malignancies. Virulence. (2017) 8:391–402. 10.1080/21505594.2016.1213472 27470662 PMC5477691

[11] Sahitya DSK Jandiyal A Jain A Senapati J Nanda S Aggarwal M Prevention and management of carbapenem-resistant Enterobacteriaceae in haematopoietic cell transplantation. Ther Adv Infect Dis. (2021) 8:20499361211053480. 10.1177/20499361211053480 34733507 PMC8558808

[12] Wu WQ Zhang YQ Xu J Tang ZX Li SJ Wei XY Risk factors for carbapenem-resistant Enterobacteriaceae colonization and the effect on clinical outcomes and prognosis in allogeneic hematopoietic stem cell transplanted patients. Infect Drug Resist. (2023) 16:6821–31. 10.2147/IDR.S424048 37904832 PMC10613414

[13] 2019 Antibiotic Resistance Threats Report. 2019 Antibiotic resistance threats report. Available online at: https://www.cdc.gov/antimicrobial-resistance/data-research/threats/index.html (Accessed January 29, 2025).

[14] World Health Organization (2017). WHO publishes list of bacteria for which new antibiotics are urgently needed. Available online at: https://www.who.int/news/item/27-02-2017-who-publishes-list-of-bacteria-for-which-new-antibiotics-are-urgently-needed (Accessed January 29, 2025).

[15] Kanate AS Majhail NS Savani BN Bredeson C Champlin RE Crawford S Indications for hematopoietic cell transplantation and immune effector cell therapy: guidelines from the American society for transplantation and cellular therapy. Biol Blood Marrow Transplant. (2020) 26:1247–56. 10.1016/j.bbmt.2020.03.002 32165328

[16] Bacigalupo A Ballen K Rizzo D Giralt S Lazarus H Ho V Defining the intensity of conditioning regimens: working definitions. Biol Blood Marrow Transplant. (2009) 15:1628–33. 10.1016/j.bbmt.2009.07.004 19896087 PMC2861656

[17] Carbapenem-resistant Enterobacterales (CRE) Infection Control. Available online at: https://www.cdc.gov/cre/hcp/infection-control/index.html (Accessed January 29, 2025).

[18] Sharland M Cappello B Ombajo LA Bazira J Chitatanga R Chuki P The WHO AWaRe antibiotic book: providing guidance on optimal use and informing policy. Lancet Infect Dis. (2022) 22:1528–30. 10.1016/S1473-3099(22)00683-1 36309019

[19] Morgan CJ. Reducing bias using propensity score matching. J Nucl Cardiol. (2018) 25:404–6. 10.1007/s12350-017-1012-y 28776312

[20] Rubin DB. Using propensity scores to help design observational studies: application to the tobacco litigation. Health Serv Outcomes Res Methodol. (2001) 2:169–88. 10.1023/A:1020363010465

[21] Haukoos JS Lewis RJ. The propensity score. JAMA. (2015) 314:1637. 10.1001/jama.2015.13480 26501539 PMC4866501

[22] D’Souza A Fretham C Lee SJ Arora M Brunner J Chhabra S Current use of and trends in hematopoietic cell transplantation in the United States. Biol Blood Marrow Transplant. (2020) 26:e177–82. 10.1016/j.bbmt.2020.04.013 32438042 PMC7404814

[23] Pan L Li J Lin Q Feng X Li X Zhang G Incidence, risk factors, and outcomes of bloodstream infection during conditioning phase before allogeneic hematopoietic stem cell transplantation. Transplant Cell. Ther. (2025) In Press. 10.1016/j.jtct.2025.05.027 40578622

[24] Pereira MR Pouch SM Scully B. Infections in allogeneic stem cell transplantation. In: Safdar A, editor. Principles and Practice of Transplant Infectious Diseases. New York, NY: Springer (2019). p. 209–26. Available online at: http://link.springer.com/10.1007/978-1-4939-9034-4_11 (Accessed October 12, 2025).

[25] Carreira AS Salas MQ Remberger M Basso IN Law AD Lam W Bloodstream infections and outcomes following allogeneic hematopoietic cell transplantation: a single-center study. Transplant Cellular Therapy. (2022) 28:50.e1–8. 10.1016/j.jtct.2021.10.008 34656808

[26] Salas MQ Charry P Puerta-Alcalde P Martínez-Cibrian N Solano MT Serrahima A Bacterial bloodstream infections in patients undergoing allogeneic hematopoietic cell transplantation with post-transplantation cyclophosphamide. Transplant Cellular Therapy. (2022) 28:850.e1–e10. 10.1016/j.jtct.2022.09.001 36089250

[27] Van Leeuwen LPM Du Toit J McMillan B Tadzimirwa GY Oosthuizen J Brown K Bloodstream infections and colonization in hematopoietic stem cell transplant recipients at a South African center: a retrospective analysis. Transplant Cellular Therapy. (2025) 31:269.e1–e13. 10.1016/j.jtct.2025.02.010 39952366

[28] World Health Organization. Global Antimicrobial Resistance and use Surveillance System (GLASS) Report 2022. Geneva: WHO (2022). Available online at: https://journals.plos.org/plosone/article/file?id=10.1371%2Fjournal.pone.0297921&type=printable (Accessed October 14, 2025).

[29] Hsu J. How COVID-19 is accelerating the threat of antimicrobial resistance. Br Med J. (2020) 369:m1983. 10.1136/bmj.m1983 32423901

[30] Tomczyk S Taylor A Brown A de Kraker MEA El-Saed A Alshamrani M Impact of the COVID-19 pandemic on the surveillance, prevention and control of antimicrobial resistance: a global survey. J Antimicrob Chemother. (2021) 76:3045–58. 10.1093/jac/dkab300 34473285 PMC8499888

[31] Slade M Goldsmith S Romee R DiPersio JF Dubberke ER Westervelt P Epidemiology of infections following haploidentical peripheral blood hematopoietic cell transplantation. Transpl Infect Dis. (2017) 19:e12629. 10.1111/tid.12629 PMC5459579 28030755

[32] Lim LM Ly N Anderson D Yang JC Macander L Jarkowski A Resurgence of colistin: a review of resistance, toxicity, pharmacodynamics, and dosing. Pharmacotherapy. (2010) 30:1279–91. 10.1592/phco.30.12.1279 21114395 PMC4410713

[33] Kaye KS Marchaim D Thamlikitkul V Carmeli Y Chiu CH Daikos G Colistin monotherapy versus combination therapy for carbapenem-resistant organisms. NEJM Evidence. (2023) 2(1). 10.1056/EVIDoa2200131 37538951 PMC10398788

[34] George B Kulkarni U Lionel S Devasia AJ Aboobacker FN Lakshmi KM Haploidentical transplantation is feasible and associated with reasonable outcomes despite major infective complications–A single center experience from India. Transplant Cellular Therapy. (2022) 28:45.e1–8. 10.1016/j.jtct.2021.09.020 34607073

[35] Esquirol A Pascual MJ Kwon M Pérez A Parody R Ferra C Severe infections and infection-related mortality in a large series of haploidentical hematopoietic stem cell transplantation with post-transplant cyclophosphamide. Bone Marrow Transplant. (2021) 56:2432–44. 10.1038/s41409-021-01328-4 34059802 PMC8165955

[36] Liu Y Liu Y Chen X Jia Y. Clinical characteristics and mortality risk factors of mixed bacterial infections in hematopoietic stem cell transplantation recipients. Front Cell Infect Microbiol. (2023) 13:1223824. 10.3389/fcimb.2023.1223824 37790911 PMC10543755

[37] Muffly L Pasquini MC Martens M Brazauskas R Zhu X Adekola K Increasing use of allogeneic hematopoietic cell transplantation in patients aged 70 years and older in the United States. Blood. (2017) 130:1156–64. 10.1182/blood-2017-03-772368 28674027 PMC5580273

[38] Weller JF Lengerke C Finke J Schetelig J Platzbecker U Einsele H Allogeneic hematopoietic stem cell transplantation in patients aged 60–79 years in Germany (1998–2018): a registry study. Haematol. (2023) 109:431–43. 10.3324/haematol.2023.283175 PMC10831926 37646665

[39] Kang SW Lee DG Kim YR Kim SJ Kim H Park J Pre-hematopoietic stem cell transplantation colonization with carbapenem-resistant enterobacterales increases the risk of subsequent bacteremia in transplant recipients. Open Forum Infect Dis. (2025) 12(9):ofaf516. 10.1093/ofid/ofaf516 40933277 PMC12418090

[40] Bilinski J Grzesiowski P Sorensen N Madry K Muszynski J Robak K Fecal microbiota transplantation in patients with blood disorders inhibits gut colonization with antibiotic-resistant bacteria: results of a prospective, single-center study. Clin Infect Dis. (2017) 65(3):364–70. 10.1093/cid/cix252 28369341

[41] Innes AJ Mullish BH Fernando F Adams G Marchesi JR Kordasti S Faecal microbiota transplantation for multi-drug resistant organisms in haematopoietic stem cell transplant recipients. Front Cell Infect Microbiol. (2021) 11:6846. 10.3389/fcimb.2021.684659

[42] U.S. Food & Drug Administration. Drug Approval Package: AVYCAZ (ceftazidime-avibactam) Injection. Application No. 206494 Approval Date: February 25, 2015. Available online at: https://www.accessdata.fda.gov/drugsatfda_docs/nda/2015/206494orig1s000toc.cfm (Accessed August 20, 2025).

[43] U.S. Food & Drug Administration. Label: VABOMERE (meropenem-vaborbactam). (2017) Application No. 209776. Available online at: https://www.accessdata.fda.gov/drugsatfda_docs/label/2017/209776lbl.pdf (Accessed October 2025).

[44] U.S. Food & Drug Administration. Label: Recarbrio (imipenem-cilastatin + relebactam). (2019). Application No. 212819. Available online at: https://www.accessdata.fda.gov/drugsatfda_docs/label/2019/212819s000lbl.pdf (Accessed October 13, 2025).

[45] Livermore DM Mushtaq S Warner M Vickers A Woodford N. *In vitro* activity of cefepime/zidebactam (WCK 5222) against gram-negative bacteria. J Antimicrob Chemother. (2017) 72:1373–85. 10.1093/jac/dkw593 28158732

[46] Rodvold KA Gotfried MH Chugh R Gupta M Patel A Chavan R Plasma and intrapulmonary concentrations of cefepime and zidebactam following intravenous administration of WCK 5222 to healthy adult subjects. Antimicrob Agents Chemother. (2018) 62:e00682–18. 10.1128/AAC.00682-18 29784852 PMC6105785

[47] Liu B Trout REL Chu GH McGarry D Jackson RW Hamrick JC Discovery of taniborbactam (VNRX-5133): a broad-Spectrum serine- and metallo-*β*-lactamase inhibitor for carbapenem-resistant bacterial infections. J Med Chem. (2020) 63:2789–801. 10.1021/acs.jmedchem.9b01518 31765155 PMC7104248

[48] Mallalieu NL Winter E Fettner S Patel K Zwanziger E Attley G Safety and pharmacokinetic characterization of nacubactam, a novel *β*-lactamase inhibitor, alone and in combination with meropenem, in healthy volunteers. Antimicrob Agents Chemother. (2020) 64:e02229–19. 10.1128/AAC.02229-19 32041717 PMC7179653

[49] Mushtaq S Vickers A Woodford N Livermore DM. Activity of nacubactam (RG6080/OP0595) combinations against MBL-producing Enterobacteriaceae. J Antimicrob Chemother. (2019) 74:953–60. 10.1093/jac/dky522 30590470

[50] Kortright KE Chan BK Koff JL Turner PE. Phage therapy: a renewed approach to combat antibiotic-resistant bacteria. Cell Host Microbe. (2019) 25(2):219–32. 10.1016/j.chom.2019.01.014 30763536

[51] Lin DM Koskella B Lin HC. Phage therapy: an alternative to antibiotics in the age of multi-drug resistance. World J Gastrointest Pharmacol Ther. (2017) 8(3):162–73. 10.4292/wjgpt.v8.i3.162 28828194 PMC5547374

[52] Aqel AA Giakkoupi P Alzoubi H Masalha I Ellington MJ Vatopoulos A. Detection of OXA-48-like and NDM carbapenemases producing Klebsiella pneumoniae in Jordan: a pilot study. J Infect Public Health. (2017) 10(2):150–5. 10.1016/j.jiph.2016.02.002 26993738

[53] Zowawi HM Sartor AL Balkhy HH Walsh TR Al Johani SM AlJindan RY Molecular characterization of carbapenemase-producing Escherichia coli and Klebsiella pneumoniae in the countries of the gulf cooperation council: dominance of OXA-48 and NDM producers. Antimicrob Agents Chemother. (2014) 58(6):3085–90. 10.1128/AAC.02050-13 24637692 PMC4068443

