# Supplementary material for: Outcomes of allogeneic hematopoietic cell transplantation in patients with carbapenem-resistant organisms infection: a propensity score-matched analysis
Source: Front Transplant. 2026 May 15;5:1818037. doi: 10.3389/frtra.2026.1818037 (PMC13219240; doi:10.3389/frtra.2026.1818037)
Supplement: Supplementary file 1 [file Datasheet1.pdf]

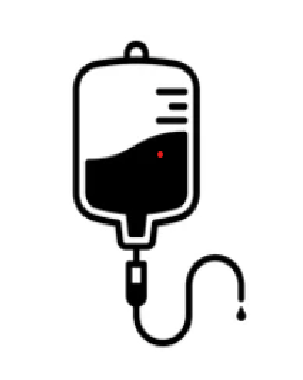

Allo-HCT recipients 2015–2024 (n=486)

CRO infection within 12 months (n=43)

Controls (n=128 after 1:3 PSM)

1:3 propensity score matching

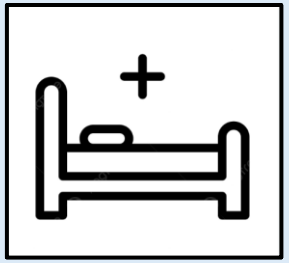

↑ ICU bed
29.2% vs 16.1%, *p*=0.047

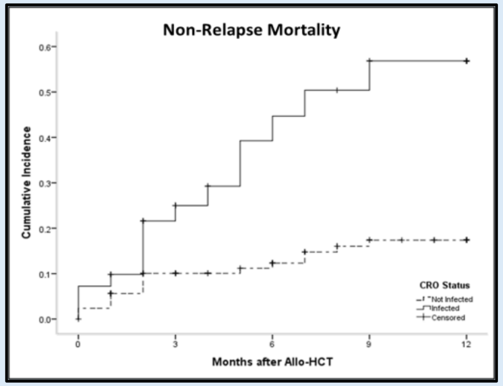

↑100 day-NRM
20.8% vs 9.1%, *p*=0.038

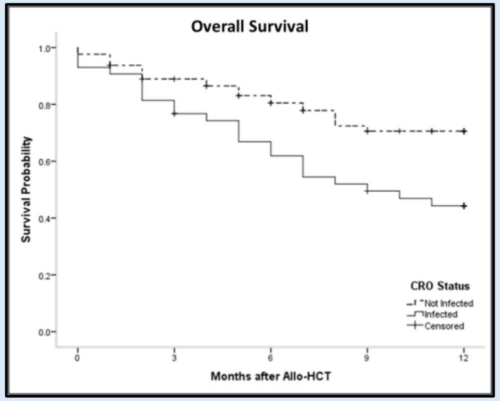

↓ 1-year OS
50% vs 74.1%, *p*=0.002

CRO infections: Independent predictor of poor outcomes

There is an urgent need for effective preventive measures, antibiotic stewardship programs, and innovative therapeutic strategies to mitigate the impact of CRO infection
