# Supplementary material for: Outcomes of allogeneic hematopoietic cell transplantation in patients with carbapenem-resistant organisms infection: a propensity score-matched analysis
Source: Front Transplant. 2026 May 15;5:1818037. doi: 10.3389/frtra.2026.1818037 (PMC13219240; doi:10.3389/frtra.2026.1818037)

| **Variable** | **Included in logistic regression model** | **Post-matching SMD** |
| --- | --- | --- |
| Age at transplant | Yes | < 0.2 |
| Gender | Yes | < 0.2 |
| Year of transplant | Yes | < 0.2 |
| Stem cell source | Yes | < 0.2 |
| Donor type | Yes | < 0.2 |
| Diagnosis | Yes | < 0.2 |

***Supplementary Table S1. Details of propensity score matching***

Matching algorithm: 1:3 nearest-neighbour matching without replacement

Balance assessment: Absolute standardized mean difference (SMD)

Threshold for acceptable balance: SMD < 0.2 for all covariates

***Figure 1S: Study flow diagram showing patient selection, exclusions, and propensity score matching.***


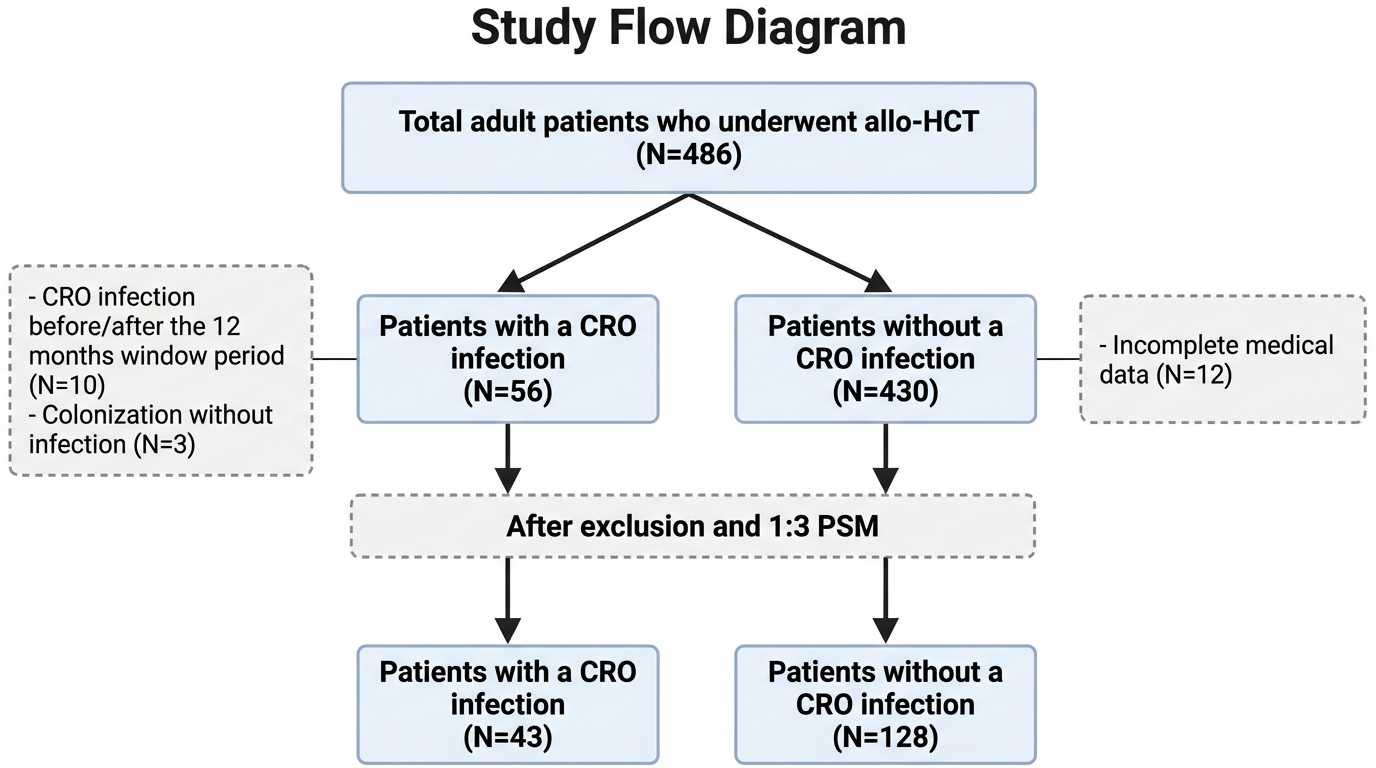

Supplement: Supplementary file 2 [file Table1.docx]
